# Prognosis of Patients With Testicular Carcinoma Is Dependent on Metastatic Site

**DOI:** 10.3389/fonc.2019.01495

**Published:** 2020-01-10

**Authors:** Peihang Xu, Jun Wang, Mierxiati Abudurexiti, Shengming Jin, Junlong Wu, Yijun Shen, Dingwei Ye

**Affiliations:** ^1^Department of Urology, Fudan University Shanghai Cancer Center, Shanghai, China; ^2^Department of Oncology, Shanghai Medical College, Fudan University, Shanghai, China

**Keywords:** metastatic testicular cancer, prognosis, metastatic site, number of metastases, SEER

## Abstract

**Background:** Existing data on the association of metastatic sites and prognosis of patients with metastatic testicular malignancy are limited. In this study, the association of survival outcome and the prognostic value of different metastatic sites in patients with metastatic testicular cancer was investigated.

**Methods:** A dataset from the Surveillance, Epidemiology and End Results (SEER) survey was selected for a retrospective metastatic testicular cancer cohort study. Patients with different metastatic sites were divided into corresponding groups for further analysis. Kaplan-Meier analysis with log-rank test was implemented for comparison of the survival distribution of cases. Multivariate Cox regression models were then applied to analyze the association of distant metastases with survival for all selected patients and subgroup based on different histological type with a single metastatic site.

**Results:** A total of 1,661 patients treated for metastatic testicular malignant tumors between 2010 to 2016 were enrolled in this cohort study. Upon initial diagnosis, 61.9, 15.2, 6.7, 6.4, and 36.2% of patients were found to have lung, liver, bone, brain, and distant lymph nodes metastatic sites, respectively. Patients with lung, liver, or bone metastases showed more undesirable prognosis for overall survival (OS) and cancer-specific survival (CSS), in contrast with those with distant lymph node metastases (all *P* < 0.05). In comparison with patients with more than one metastatic site, those with a single metastasis had extended OS and CSS (both *P* < 0.001). In patients with a single metastatic site, Kaplan-Meier analysis and multivariate Cox regression demonstrated the association of bone and liver with the worst two groups of OS and CSS. Multivariate Cox models based on histological type showed different prognostic values of metastases in patients with seminoma or non-seminomatous germ cell tumors.

**Conclusion:** There is much heterogeneity in the oncological outcome of site-specific metastatic patients. Metastatic profiles and the prognostic value of metastases are dependent on the histological type in TC patients. Distant lymph nodes and lung metastases indicate favorable prognostic factors, while bone and liver metastases indicate negative survival outcomes in TC.

## Introduction

Testicular cancer (TC), which represents 1% of male neoplasms and 5% of urological tumors, is the most common solid cancer in men between 20 and 45 years old (1, 2). With over 9,560 new cases diagnosed each year in the United States (US), the incidence of TC has been on the rise for the last 10 years, especially in more economically-developed areas (2–5). For metastatic TC (mTC), the treatment load of platinum-based chemotherapy is usually associated with cure rate. Inappropriate systemic treatment can result in a low rate of cure, whereas overtreatment may lead to acute and late adverse events (6, 7). In this regard, characterizing mTC into different groups based on variant clinical outcome is indispensable.

Many factors have been demonstrated as contributing to the prognosis of advanced TC, and metastatic site has an important role in many malignancies (8, 9). The favorable prognostic impact of lung is widely known based on the data from the International Germ Cell Cancer Collaborative Group (IGCCCG) (10). However, several drawbacks limit the generalizability of previous data in contemporary populations. For example, historical cohorts were evaluated in this study, and only a small proportion of the patients received the current standard chemotherapy (10). Furthermore, despite TC tending to spread to sites including lung and distant lymph nodes, the risk of atypical metastases is not negligible (10, 11). Approximately 9–11% of the patients with mTC have atypical metastases (non-pulmonary) (10, 11). However, little is known about the prognostic value of non-pulmonary metastases since limited data exists on the previous findings (10–15). Consequently, to address this lack of knowledge and to investigate the potential prognostic value of site-specific metastases in TC, we studied the association of survival outcome and prognostic value with different metastatic sites in patients with metastatic cancer in a retrospective metastatic TC cohort from the US.

## Methods

### Cohort Selection and Data Collection

A retrospective metastatic TC cohort including patients with primary mTC (M1 stage) from 2010 to 2016 was selected from the Surveillance, Epidemiology and End Results (SEER) program dataset. Additional inclusion criteria were as follows: patients must be no <14 years old of age; TC was the primary diagnosis of cancer; distant metastatic sites must be documented, including distant lymph nodes, lung, liver, bone, and brain; patient received active follow-up; and patients had a survival length ≥ 1 month. We excluded patients with insufficient data of pathological characteristics, distant metastases, M1 stage, extragonadal germ cell tumor, and data of survival outcomes. Patients with controversial data (e.g., patients at M1a stage without metastases of lung or distant lymph nodes) were also excluded from the cohort (Figure S1). Covariates such as metastatic sites, surgery of primary tumor, TNM stage, serum tumor markers after orchiectomy, and pathological characteristics were included in the analysis. Survival time (months), the status of survival, and cause of death were also included in the analysis. S stage was obtained according to different level of patient's postoperative tumor markers including alpha fetoprotein (AFP), human chorionic gonadotropin (hCG), and lactate dehydrogenase (LDH) based on the staging system of the 2016 Tumor, Node, Metastasis (TNM) of the International Union Against Cancer (UICC). Data such as systemic therapy, radiation therapy, and surgery of metastatic site were not available in the database. Unless specifically emphasized, distant lymph nodes generally refer to following three conditions: (1) retroperitoneal lymph nodes specified as above the diaphragm; (2) external iliac nodes, pelvic nodes and inguinal nodes without or unknown previous scrotal or inguinal surgery prior to presentation of the testis tumor; (3) other kinds of metastatic distant lymph nodes. It is worth noting that external iliac nodes, pelvic nodes as well as inguinal nodes with previous scrotal or inguinal surgery prior to presentation of the testis tumor were classified as regional nodes. And retroperitoneal nodes below the diaphragm, nodes around spermatic vein, periaortic nodes, and pericaval nodes were also classified as regional lymph nodes.

### Study Outcomes

Overall survival (OS) and cancer-specific survival (CSS) were the two major outcomes of this study. OS was defined as the interval of time from TC diagnosis to death. CSS was defined as the interval from the date of TC diagnosis to death due to TC or other causes.

### Statistical Analysis

Demographic factors were reported as mean, median, and interquartile range for continuous variables, and frequency and proportion for categorical variables. Comparisons of means and proportions were analyzed by the Student's *t*-test and Chi-squared test, respectively. Survival was analyzed by the Kaplan-Meier survival analysis. Breslow test as well as log-rank test were performed to analyze the discrepancies between OS and CSS. A Cox proportional model with hazard ratios and 95% CI was then implemented for multivariate analyses of the cohort. All statistical analyses were performed using SPSS Statistics software version 20.0 (IBM, NY, US).

## Results

### Demographic and Clinical Characteristics

Overall, 1,661 patients with mTC were enrolled in the current study, and were identified according to our defined inclusion and exclusion criteria (Figure S1). Patients' demographical characteristics are shown in Table 1. The average age at diagnosis was 33.2 years, with a median of 31 years. There was no significant difference in the distribution of the number of patients in each year (from 2010 to 2016). Most of the population (90.4%) was ethnically white. More than two-thirds of the patients were unmarried. Primary TC on the right side was more common than on the left side. Approximately 84.3% of patients underwent radical orchiectomy. Patients' clinicopathological information are also summarized in Table 1 according to histological type. Since the number of patients (*n* = 80) with non-germ cell testicular cancer (NGCTC) was too small, these individuals were not listed separately in Table 1. It is notable that patients with seminoma were older than those with non-seminomatous germ cell tumor (NSGCT) (*P* < 0.001).

**Table 1:** Characteristics of patients and metastatic sites.

**Characteristics**	**Total (*n* = 1,661)**	**Seminoma (*n* = 403)**	**NSGCT (*n* = 1,178)**
Age at diagnosis, years			
Mean (median)	33.2 (31)	40.7 (40)	30.6 (28)
IQR	24–40	32–50	23–36
Year of diagnosis			
2010	213 (12.9)	42 (10.4)	165 (14.0)
2011	235 (14.1)	71 (17.6)	152 (12.9)
2012	238 (14.3)	63 (15.6)	167 (14.2)
2013	278 (16.7)	80 (19.9)	181 (15.4)
2014	240 (14.4)	63 (15.6)	164 (13.9)
2015	229 (13.8)	42 (10.4)	173 (14.7)
2016	228 (13.7)	42 (10.4)	176 (14.9)
Race			
White	1,501 (90.4)	367 (91.1)	1,066 (90.5)
Black	58 (3.5)	16 (4.0)	40 (3.4)
Others	91 (5.5)	16 (4.0)	65 (5.5)
Unknown	11 (0.7)	4 (1.0)	7 (0.6)
Marital status			
Married	459 (27.6)	160 (39.7)	274 (23.3)
Unmarried	1,140 (68.6)	226 (56.1)	863 (73.3)
Unknown	62 (3.7)	17 (4.2)	41 (3.5)
T stage			
T0	72 (4.3)	37 (9.2)	33 (2.8)
T1	534 (32.1)	119 (29.5)	390 (33.1)
T2	444 (26.7)	74 (18.4)	337 (28.6)
T3	281 (16.9)	42 (10.4)	233 (19.8)
T4	39 (2.3)	14 (3.5)	23 (2.0)
Tx	291 (17.5)	117 (29.0)	162 (13.8)
N stage			
N0	516 (31.1)	125 (31.0)	356 (30.2)
N1	436 (26.2)	94 (23.3)	323 (27.4)
N2	239 (14.4)	32 (7.9)	197 (16.7)
N3	365 (22.0)	120 (29.8)	232 (19.7)
Nx	105 (6.3)	32 (7.9)	70 (5.9)
S stage			
S0	146 (8.8)	38 (9.4)	103 (8.7)
S1	210 (12.6)	31 (7.7)	173 (14.7)
S2	189 (11.4)	32 (7.9)	145 (12.3)
S3	179 (10.8)	28 (6.9)	137 (11.6)
Sx	937 (56.4)	274 (68.0)	620 (52.6)
Laterality			
Left-origin of primary	725 (43.6)	165 (40.9)	527 (44.7)
Right-origin of primary	812 (48.9)	177 (43.9)	593 (50.3)
Bilateral, single primary	4 (0.2)	0 (0.0)	3 (0.3)
unknown	120 (7.2)	61 (15.1)	55 (4.7)
Radical orchiectomy			
Yes	1,401 (84.3)	296 (73.4)	1,034 (87.8)
No	243 (14.6)	102 (25.3)	132 (11.2)
Unknown	17 (1.0)	5 (1.2)	12 (1.0)

*IQR, interquartile range; NSGCT, non-seminomatous germ cell tumor*.

### Distribution of Distant Metastases

**Figure 1:**
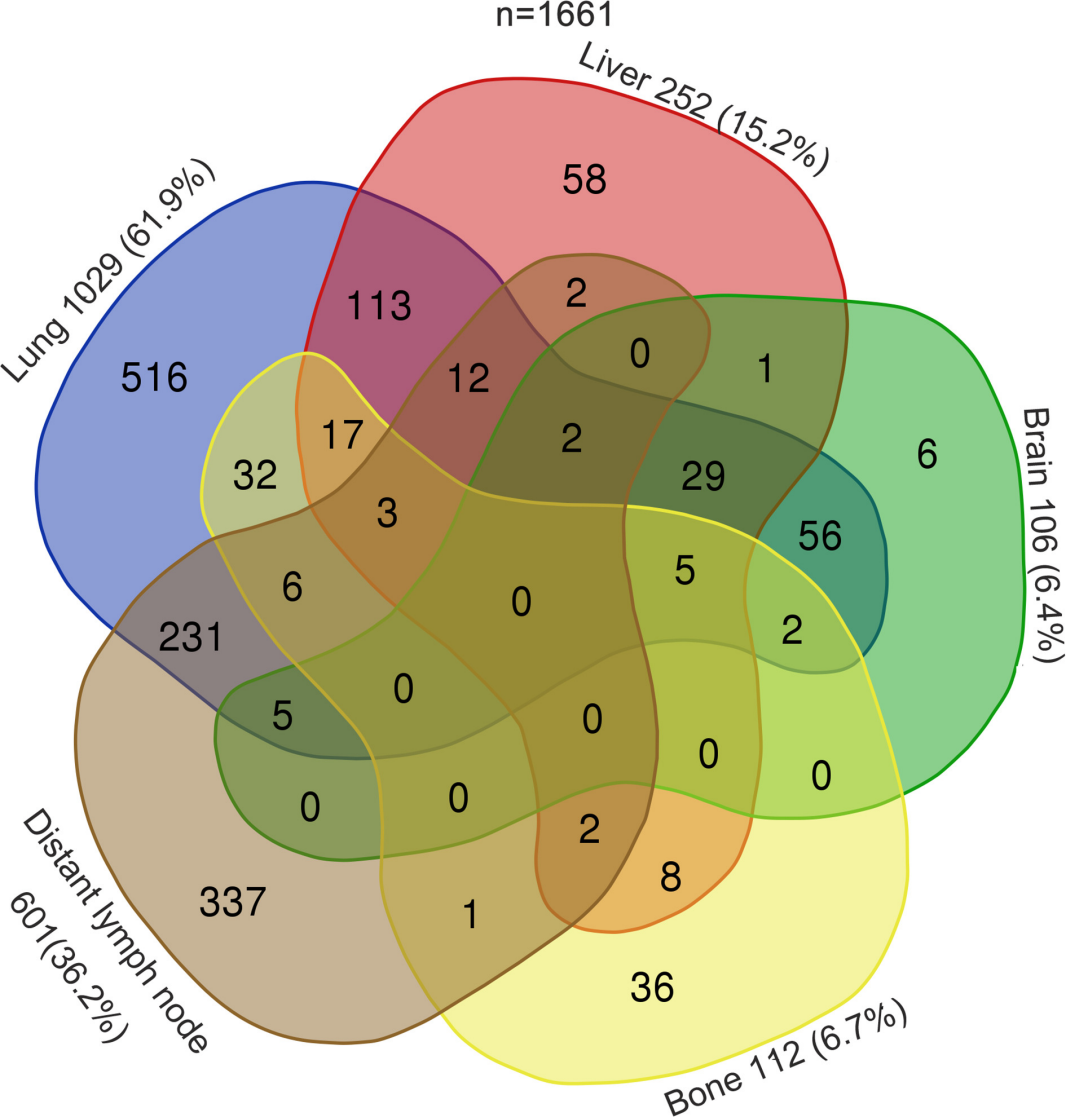
Venn diagram of the distribution of distant metastatic sites in the overall cohort.

The distributions and discrepancies in the sites of metastases of TC patients are illustrated in the Venn diagram of Figure 1. The most common sites of metastases were lung (1,029 cases, 61.9%), followed by distant lymph nodes (601 cases, 36.2%), liver (252 cases, 15.2%), bone (112 cases, 6.7%), and brain (106 cases, 6.4%). Most patients (953, 57.4%) had a single site of distant metastases, followed by two sites (444, 26.7%), three sites (73, 4.4%), and four sites (10, 0.6%). No more than four metastatic sites were found in any one case of the cohort. When classified according to histological type, the distribution was different than that of metastases in TC patients (Figures S2A, S2B). Patients with seminoma usually had distant lymph node metastases (199 cases, 49.4%), followed by that to lung (108 cases, 26.8%) (Figure S2A). Furthermore, patients with NSGCT were more likely to have metastases to lung (855 cases, 72.5%), followed by distant lymph nodes (385 cases, 32.6%) (Figure S2B).

### Associations Between Metastatic Sites and Survival Outcomes

**Figure 2:**
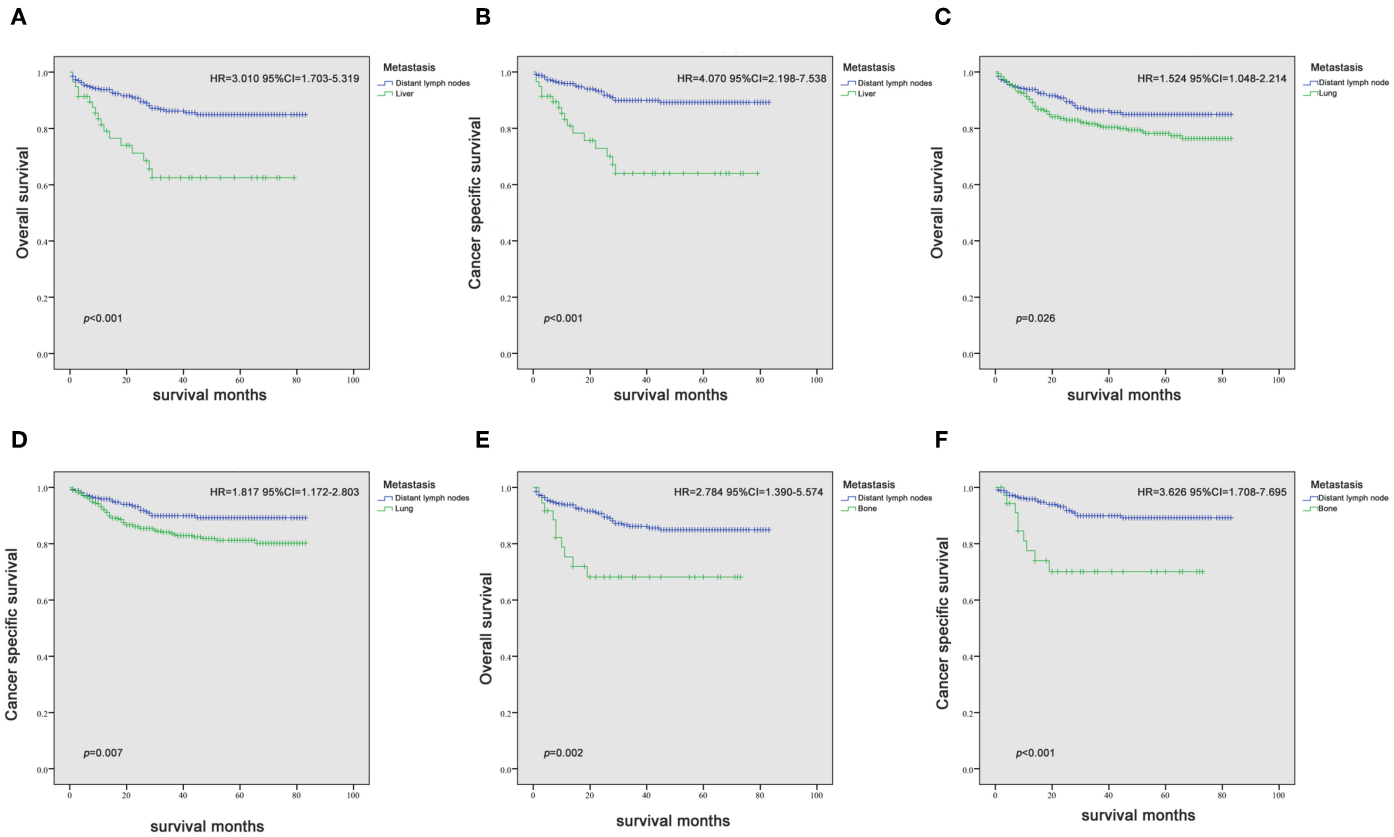
Kaplan-Meier curves of overall survival and cancer-specific survival in patients with liver **(A,B)**, lung **(C,D)**, and bone **(E,F)** metastases vs. patients with distant lymph node metastasis.

**Figure 3:**
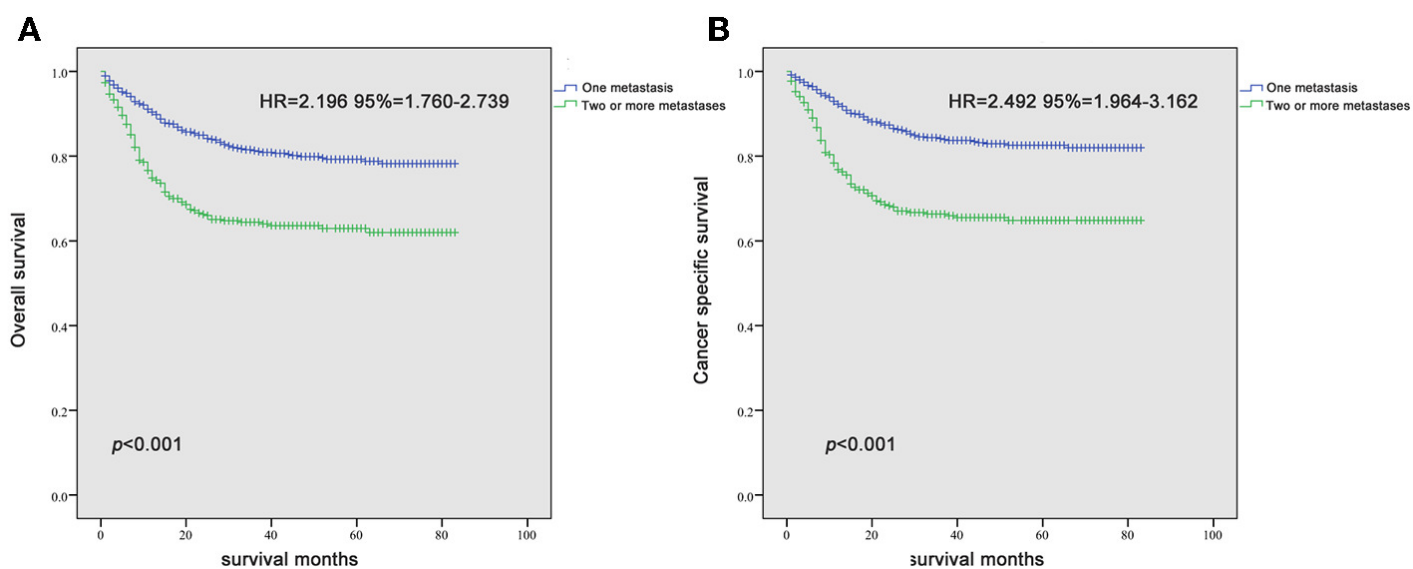
Kaplan-Meier curves of overall survival **(A)** and cancer-specific survival **(B)** according to the number of metastatic sites.

**Figure 4:**
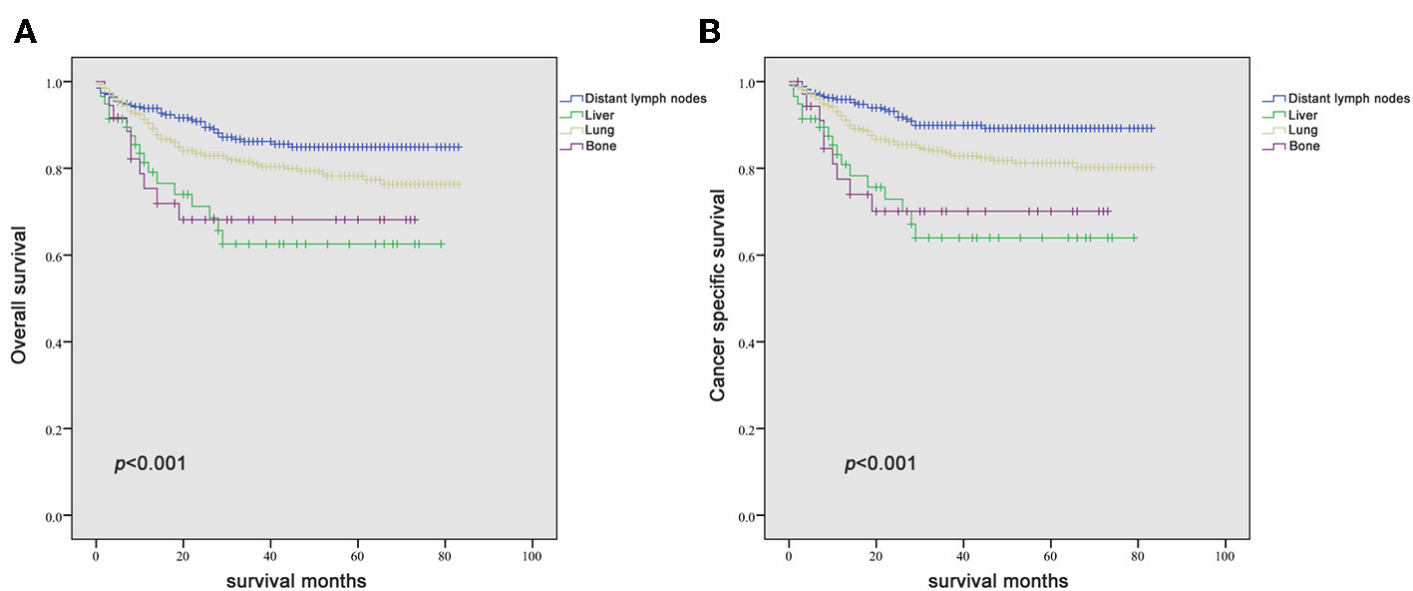
Kaplan-Meier curves of overall survival **(A)** and cancer-specific survival **(B)** according to the site of metastasis in patients with a single metastatic site.

Since it is commonly acknowledged that patients with distant lymph node metastases have favorable prognoses, we took distant lymph node metastases as a reference and patients with a single metastatic site were included in the Kaplan-Meier analysis (*n* = 947). Patients with brain metastases were excluded in the analysis of patients with only one metastasis due to insufficient samples (only six patients). In Figures 2A–F, it was consistent with clinical experience that patients diagnosed with liver, lung, and bone metastases had worse outcomes in the measurement of both OS and CSS, compared with those with distant lymph node metastases alone. Using log-rank test to compare the number of metastatic sites in all patients (*n* = 1,661) with TC, significantly longer OS and CSS was observed in patients with one metastatic site than those with more metastases (OS: HR = 2.196, 95% CI = 1.760–2.739; CSS: HR = 2.492, 95% CI = 1.964–3.162; both *P* < 0.001) (Figures 3A,B) In the subgroup of patients with only one metastatic site (*n* = 947), patients with lymph node and lung metastases showed significantly longer OS and CSS compared with bone and liver metastases by log-rank comparison (*P* < 0.001) (Figures 4A,B). Since curves of liver metastases and bone metastases crossed with each other in the Kaplan-Meier analysis, comparisons between liver and bone metastases required further multivariable Cox regression analysis.

### Multivariable Cox Regression Models

Parameters besides histology, T stage, N stage, S stage, and site of metastases were included in the multivariable Cox regression model of single-site metastasis patients. As shown in Table 2, histology, T stage, and metastatic site were independent prognostic factors for mTC patients both in OS and CSS (all *P* < 0.05). S stage was a prognostic factor only for CSS (*P* = 0.016). However, it still showed a trend in predicting OS (*P* = 0.052). N stage showed no statistical difference in predicting OS and CSS of mTC patients. In the analysis of metastatic sites, bone and liver represented the two groups with the worst prognosis of OS and CSS (all *P* < 0.05) (Table 2). Lung and brain metastases did not achieve statistical difference.

**Table 2:** Multivariable Cox regression analysis predicting overall survival and cancer-specific-survival in 953 patients diagnosed with a single metastatic site within the SEER database between 2010 and 2016.

**Characteristics**	**Overall survival**		**Cancer specific survival**	
	**HR (95% CI)**	***p*-value**	**HR (95% CI)**	***p*-value**
Histology		0.001		<0.001
Seminoma	Reference		Reference	
NSGCT	1.410 (0.919–2.163)	0.115	1.817 (1.102–2.999)	0.019
NGCTC	3.578 (1.856–6.894)	<0.001	4.958 (2.421–10.155)	<0.001
T stage		0.009		0.013
T0–T2	Reference		Reference	
T3–T4	1.416 (0.943–2.127)	0.094	1.452 (0.925–2.279)	0.105
Tx	1.915 (1.123–2.967)	0.004	1.973 (1.222–3.187)	0.005
N stage		0.850		0.707
N0	Reference		Reference	
N1–N2	1.029 (0.701–1.510)	0.886	0.858 (0.561–1.312)	0.478
N3	1.197 (0.767–1.871)	0.429	1.119 (0.689–1.817)	0.650
Nx	0.960 (0.440–2.092)	0.918	1.066 (0.483–2.350)	0.875
S stage		0.052		0.016
S0	Reference		Reference	
S1	1.697 (0.696–4.136)	0.245	0.846 (0.283–2.527)	0.764
S2	1.874 (0.761–4.613)	0.172	1.947 (0.745–5.090)	0.174
S3	3.352 (1.423–7.893)	0.006	3.228 (1.280–9.142)	0.013
Sx	2.169 (1.001–4.698)	0.050	2.113 (0.916–4.874)	0.080
Site of metastases		0.029		0.004
Lung only	1.312 (0.868–1.983)	0.198	1.417 (0.880–2.281)	0.151
Liver only	2.265 (1.238–4.145)	0.008	2.843 (1.473–5.487)	0.002
Bone only	2.419 (1.190–4.919)	0.015	3.074 (1.424–6.636)	0.004
Brain only	1.622 (0.221–11.913)	0.635	2.642 (0.354–19.691)	0.343
Distant nodes only	Reference		Reference	

*HR, hazard ratio; NSGCT, non-seminomatous germ cell tumor; NGCTC, non-germ cell testicular cancer*.

### Subgroup Analysis Based on Histological Type

Among mTC patients with single metastases, a total of 251 patients with seminoma and 655 patients with NSGCT were included in the subgroup analysis, while patients with brain metastases were excluded (*n* = 6).

**Figure 5:**
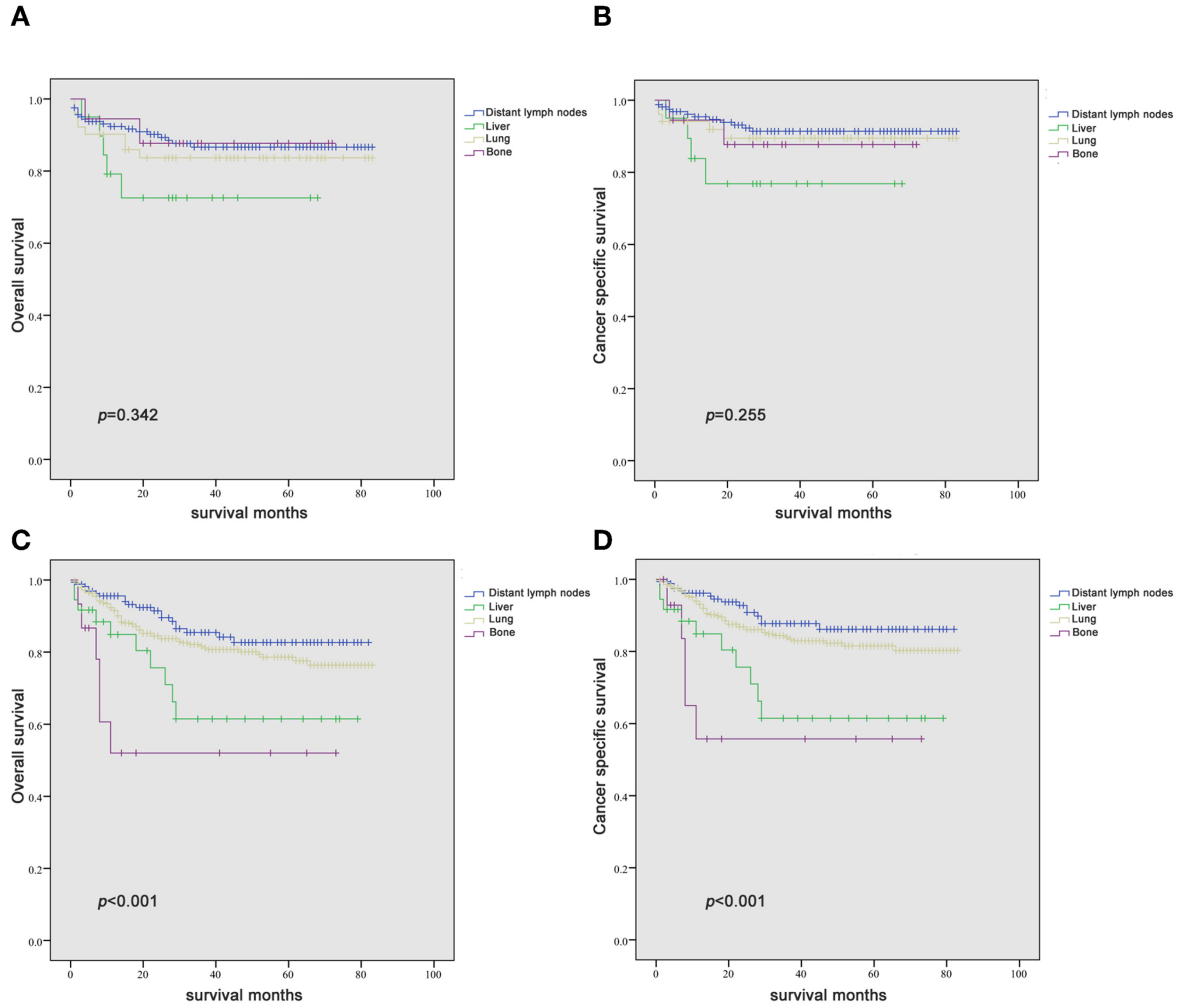
Kaplan-Meier curves of overall survival and cancer-specific survival in patients with seminoma **(A,B)** and non-seminomatous germ cell tumor **(C,D)** according to the site of metastasis in patients with a single metastatic site.

For patients with NSGCT, bone and liver metastases showed worse outcome than lung and distant lymph node metastases both in OS and CSS (both *P* < 0.001) (Figures 5C,D). The same results could be validated in multivariable Cox models (Tables 3, 4). Especially, bone metastases showed a worst outcome compared with the other three types of metastases (OS: HR = 5.116, 95% CI = 2.031–13.884; CSS: HR = 5.243, 95% CI = 1.900–14.466; both *P* = 0.001).

**Table 3:** Multivariable Cox regression analysis predicting overall survival in patients of different histological type diagnosed with a single metastatic site within the SEER database between 2010 and 2016.

**Characteristics**	**Seminoma**		**NSGCT**	
	**HR (95% CI)**	***p*-value**	**HR (95% CI)**	***p*-value**
T stage		0.096		0.038
T0–T2	Reference		Reference	
T3–T4	1.648 (0.627–4.328)	0.311	1.285 (0.792–2.085)	0.309
Tx	2.353 (1.079–5.130)	0.031	2.024 (1.168–3.506)	0.012
S stage		0.715		0.108
S0	Reference		Reference	
S1	4.727 (0.525–42.563)	0.166	1.274 (0.470–3.453)	0.634
S2	2.690 (0.241–30.004)	0.421	1.571 (0.580–4.256)	0.375
S3	3.694 (0.370–36.888)	0.266	2.892 (1.140–7.339)	0.025
Sx	2.994 (0.400–22.417)	0.286	1.650 (0.708–3.849	0.246
Site of metastases		0.453		0.004
Lung only	1.206 (0.522–2.787)	0.661	1.321 (0.794–2.198)	0.283
Liver only	2.176 (0.783–6.050)	0.136	1.922 (0.864–4.273)	0.109
Bone only	0.735 (0.169–3.202)	0.681	5.116 (2.031–13.884)	0.001
Distant nodes only	Reference		Reference	

*HR, hazard ratio; NSGCT, non-seminomatous germ cell tumor*.

**Table 4:** Multivariable Cox regression analysis predicting cancer-specific-survival in patients of different histological type diagnosed with a single metastatic site within the SEER database between 2010 and 2016.

**Characteristics**	**Seminoma**		**NSGCT**	
	**HR (95% CI)**	***p*-value**	**HR (95% CI)**	***p*-value**
T stage		0.209		0.049
T0–T2	Reference		Reference	
T3–T4	1.108 (0.297–4.131)	0.879	1.353 (0.802–2.284)	0.257
Tx	2.182 (0.891–5.345)	0.088	2.046 (1.135–3.687)	0.017
S stage		0.820		0.083
S0	Reference		Reference	
S1	1.001 (0.997–1.211)	0.981	0.830 (0.253–2.725)	0.759
S2	2.6787 (0.238–30.098)	0.425	1.720 (0.587–5.046)	0.323
S3	3.922 (0.388–39.670)	0.247	2.844 (1.026–7.883)	0.044
Sx	2.156 (0.281–16.523)	0.460	1.737 (0.689–4.381)	0.242
Site of metastases		0.492		0.005
Lung only	1.184 (0.411–3.407)	0.754	1.359 (0.772–2.393)	0.287
Liver only	2.553 (0.777–8.391)	0.123	2.379 (1.033–5.482)	0.042
Bone only	1.122 (0.245–5.145)	0.882	5.243 (1.900–14.466)	0.001
Distant nodes only	Reference		Reference	

*HR, hazard ratio; NSGCT, non-seminomatous germ cell tumor*.

For patients with seminoma, although there was no statistical difference in the Kaplan-Meier analysis, it still showed a certain trend that liver metastases indicated the worst outcome than the other three types of metastases both in OS and CSS (Figures 5A,B). Subsequent Cox models showed the same trend, although they also did not meet statistical significance (Table 4). Interestingly, patients with bone metastases show similar survival trend as lung and lymph node metastases.

## Discussion

Prognostic system of testicular cancer proposed by IGCCCG is widely used. However, data of the IGCCCG (the largest available dataset) were accumulated from 1975 to 1990, and only a small proportion of patients received a bleomycin, etoposide, and cisplatin (BEP)-based regimens. Currently, BEP-based regimen is the main standard chemotherapy for advanced TC, which is superior to other combinations of chemotherapy agents (16). Therefore, many researches questioned the prognostic value of IGCCCG model in current population. Up to now, few studies have systematically focused on the association between metastatic sites and survival in the post-BEP era (12, 17). Our study is the first to comprehensively address the role of the metastatic site on TC patients' survival using a contemporary cohort with a large sample size. We found different metastatic profiles exist in mTC patients. Patients with seminoma tend to metastasize to distant nodes, while patients with NSGCT often develop pulmonary metastases. Notably, our study provided additional information on site-specific survival for distant metastases.

Previous studies about the impact of metastatic sites on prognosis of mTC often enrolled all metastatic populations and impact of different metastatic sites on survival often affect each other (10, 18). Since many people involves in multiple metastatic sites, it is difficult to accurately describe the impact of metastatic sites on survival.

Therefore, for the first time we conducted further analyses using a subgroup of patients with a single metastatic site. We revealed that bone and liver metastases represent the two worst survival outcomes in all mTC patients, while lung and distant nodes metastases indicate better survival than other kind of metastases. We attributed the lack of statistical difference in patients with brain metastases to the sample size (*n* = 6). However, categorization of the patients according to histological type resulted in different observations. For patients with NSGCT, distant lymph node and lung metastases showed the best two outcomes compared with bone and liver metastases, in line with the IGCCCG database. However, for patients with seminoma, bone metastases showed a similar survival trend compared with lung and distant lymph node metastases. Liver tends to bare the worst survival outcome alone. Interestingly, subgroup analysis of IGCCCG also reveal a higher 5-year survival rate of patients of seminoma with bone metastases than that of lung (10). Consequently, we suppose that the prognosis of site-specific metastases should be carefully revalued in the contemporary cohort according to histological type. In this study, we also found that more sites of metastases were associated with poorer clinical outcomes, which is easy to understand. A greater number of metastatic sites commonly suggest more aggressive biological behavior of the tumor and worse physical conditions.

Several clinical implications should be highlighted in the current study. On one hand, our results might inspire physicians to develop novel therapeutic approaches through a better understanding of the natural history of TC. On the other hand, our findings highlight the need to improve the risk stratification of metastatic patients for identifying candidate for clinical trials (19, 20).

Despite our current study identifying the association of prognostic outcomes with different metastatic sites in patients with metastatic cancer in a large retrospective TC cohort, it does have some limitations. First, the retrospective nature of our datasets may lead to incomplete or even contradictory clinical information. Second, our analyses were prevented from adjusting for potential confounders due to the lack of information regarding systemic treatment regimens or surgery toward metastatic sites, which may impact prognosis of patients (21).

## Conclusion

There is much heterogeneity regarding oncological outcomes in site-specific metastatic patients. Different metastatic profiles and different prognostic values of metastases exist in TC patients depending on histological type. Distant lymph nodes and lung metastases are favorable prognostic factors, while liver metastases indicate negative survival outcomes in TC.

## Data Availability Statement

Publicly available datasets were analyzed in this study. This data can be found here: https://seer.cancer.gov/.

## Ethics Statement

The study was approved by the Ethics Committee of Fudan University Shanghai Cancer Center. As this is a retrospective study in nature, the informed consent was not required in this study.

## Author Contributions

PX and JWa contributed to data acquisition and statistical analysis and prepared the manuscript. MA, JWu, and SJ drafted this manuscript. YS and DY supervised the study. All authors read and approved the final manuscript.

### Conflict of Interest

The authors declare that the research was conducted in the absence of any commercial or financial relationships that could be construed as a potential conflict of interest.

## Supplementary Material

Figure S1 Flow chart of inclusion and exclusion of patients. SEER, Surveillance, Epidemiology, and End Results.

Figure S2 **(A)** Venn diagram of the distribution of distant metastatic sites in patients with seminoma. **(B)** Venn diagram of the distribution of distant metastatic sites in patients with non-seminomatous germ cell tumor.
