# Supplementary figures and images for: Prognosis of Patients With Testicular Carcinoma Is Dependent on Metastatic Site
Source: Front Oncol. 2020 Jan 10;9:1495. doi: 10.3389/fonc.2019.01495 (PMC6966605; doi:10.3389/fonc.2019.01495)

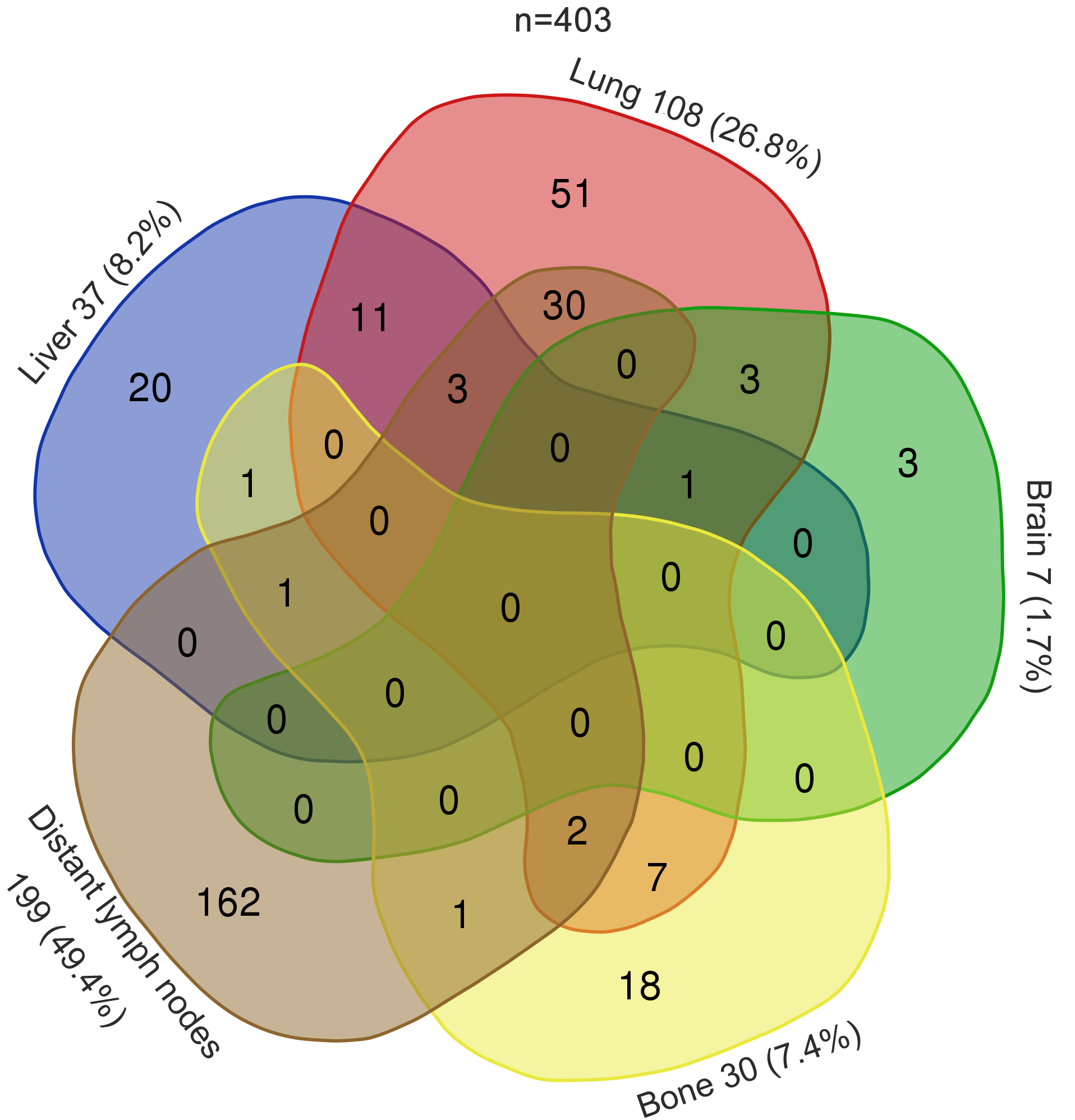

Supplement: Figure S1 — Flow chart of inclusion and exclusion of patients. SEER, Surveillance, Epidemiology, and End Results. [file Data_Sheet_1.zip › Image 2.TIF]

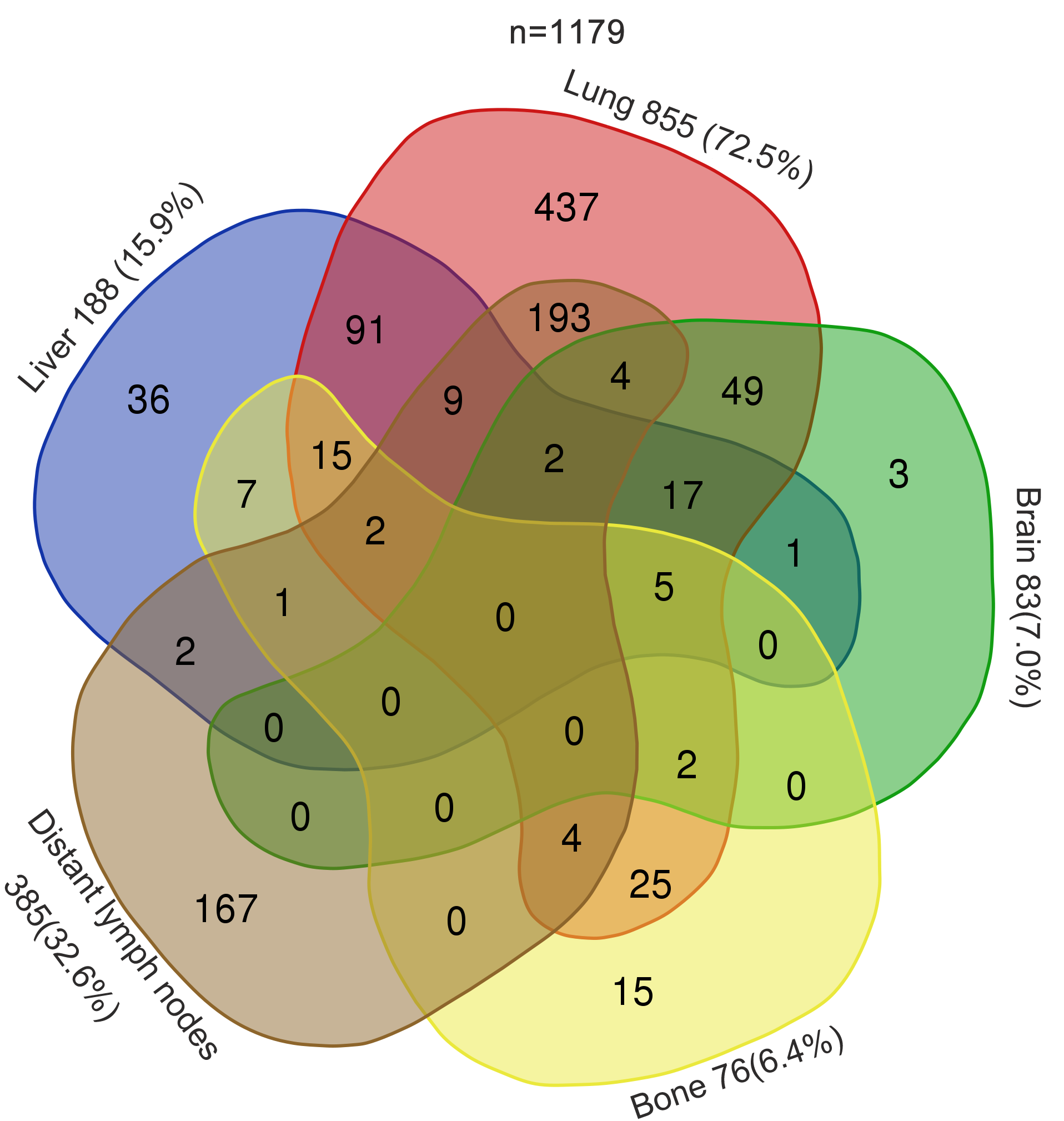

Supplement: Figure S1 — Flow chart of inclusion and exclusion of patients. SEER, Surveillance, Epidemiology, and End Results. [file Data_Sheet_1.zip › Image 3.TIF]
